# Dental injury in anaesthesia: a tertiary hospital’s experience

**DOI:** 10.1186/s12871-018-0569-6

**Published:** 2018-08-16

**Authors:** Yanni Tan, Nivan Loganathan, Kyu Kyu Thinn, Eugene Hern Choon Liu, Ne-Hooi Will Loh

**Affiliations:** 1 Department of Anaesthesia, National University Hospital Singapore, 5 Lower Kent Ridge Road, Singapore, 119074 Singapore; 2 Department of Anaesthesia, Ng Teng Fong General Hospital, 1 Jurong East Street 21, Singapore, 609606 Singapore

**Keywords:** Anaesthesia, Perioperative dental injury, Complication, Risk factors for dental injury

## Abstract

**Background:**

Dental injury is a common perioperative complication, but there are no country specific data available, especially with the use of supraglottic airway devices (SAD). The aims of our study are to report the incidence, risk factors, and local practices in the management of perioperative dental injuries in Singapore.

**Methods:**

We analyzed data from the departmental database from 2011 to 2014, noting the anticipated difficulty of airway instrumentation, intubation grade, pre-existing dental risk factors, location of dental trauma discovery, position of teeth injured and presence of dental referral. The risk factors for dental trauma were then identified using logistic regression (between 51 dental trauma patients and 55,107 patients without dental trauma).

**Results:**

The rate of dental injury was 0.092% for general anaesthesia cases. The most significant patient risk factor is the presence of pre-existing dental risk factors (OR 12.55). Anaesthetic risk factors include McGrath MAC usage (OR 2.51) and a Cormack and Lehane grade of 3 or more (OR 7.25). Most of the dental injuries were discovered in the operating theatre. 7 (13.7%) patients had SAD inserted and only 23 (45.1%) cases were referred to dental services.

**Conclusion:**

Videolaryngoscopy with the McGrath MAC is associated with an increased likelihood of dental injury. This could be either because videolarygoscopes were used when increased risk of dental trauma was anticipated, or due to incorrect technique of laryngoscopy. Future studies should be done to establish the causality. The management of dental injuries could be improved with development of departmental guidelines.

## Background

Dental injury is a common perioperative potential complication, with an incidence between 0.02–0.07% from retrospective studies [1–5]. Prospective studies have reported a higher incidence of 12.1–25.0% [6, 7]. Perianaesthetic dental trauma makes up one-third of all medico-legal claims related to anesthesia, making it the most common medico-legal complaint [5].

Dental injuries include enamel fractures, loosened or subluxated teeth, tooth avulsion, crown or root fracture. Newland et al. reported that the anaesthetist detected 86% of all dental injuries, while only 14% were reported by the patient [2].

Dental trauma management has been described to include accounting for all dental fragments, offering a full explanation and clear apology to the patient when sufficiently awake, and organizing urgent dental assessment [8].

While there are several studies reporting the incidence and risk factors associated with dental trauma, there are no local data available [1–5]. Furthermore, no studies have reported the incidence with the use of supraglottic airway devices. The primary objective of our study is to report the local incidence and risk factors of perioperative dental injuries, while the secondary aim is to identify the local practices in the management of such complication and their impact on outcome.

## Methods

Approval for the study as well as waiver for informed consent was obtained from the institutional review board (IRB) of National University Hospital (NUH), National Healthcare Group (NHG), Singapore. We analyzed data retrospectively from the departmental anaesthesia audit database from January 2011 through December 2014. The database contained information regarding patient biodata, type of surgery, surgical discipline, comorbidities, airway assessment, type of airway used, type of anaesthetics, timing and duration of surgery, significant perioperative events and presence of any critical incidents. Dental trauma was listed as a procedural complication. Three independent anaesthetists were involved in collating the data. Information from the database was cross-referenced with electronic patient medical records. We noted any features of anticipated difficult airway, intubation grade, pre-existing dental risk factors, location of dental trauma discovery, position of teeth injured, presence of dental referral and outcomes, and analysed these factors using a combination of chi-square test and logistic regression. Three types of supraglottic airway devices were used in the institution during the review period: the ProSeal LMA, Supreme LMA and I-gel. All videolaryngoscopy was done using the McGrath MAC laryngoscope and number of McGrath blades used was taken as a surrogate of the incidence of videolaryngoscopies performed.

The known risk factors were compared between the 51 dental trauma patients and the 55,107 patients without dental trauma. The data was analyzed with IBM SPSS Version 22, using the Chi-square test for categorical variables. Logistic regression was also used to calculate the Odds Ratio (OR) with *P* values for significance and 95% confidence intervals. Spearman correlation was adopted to associate age and ASA while Pearson’s correlation was used to associate age and poor dentition.

## Results

There were a total of 78,682 records on the database during this four-year period; of which there were 55,158 general anaesthesia cases with airway manipulation, and 51 cases of dental injuries. The rate of dental injury was 0.092% for all general anaesthesia cases and 0.065% of all anaesthetic techniques.

Regarding pre-existing dental risk factors, 26 (51%) had loose tooth/teeth; 14 (27.5%) had periodontal disease or poor oral hygiene; 7 (13.7%) had dental prosthesis (i.e. crowns, bridges or implants). Majority of described loose dentition or prosthesis that were damaged also had periodontal disease and poor oral hygiene. 3 patients had incomplete data.

Table 1 tabulates the risk factors with OR comparing patients with dental trauma to the control group. The most significant patient risk factor is poor pre-existing dentition (OR 12.55), followed by reduced thyromental distance and Mallampati score of more than 3. Other difficult airway predictors such as reduced mouth opening and abnormal neck extension were not significant although there was a tendency towards positive correlation. Overall, anticipated difficult airway by the anaesthetist has an OR of 3 (*p* < 0.001). ASA status of 3 or more also has an associated increased risk of dental injury.

**Table 1:** Risk factors associated with dental injury

Risk factors	Cases (*n* = 51)	Controls (*n* = 55107)	*P*	OR	95% CI
Emergency surgery	12	10873	NS	0.8	0.42–1.53
ASA ≥ 3	20	10648	0.001	2.44	1.39–4.29
Anticipated difficult airway	14	4935	< 0.001	3.00	1.62–5.55
Mallampatti					
≥ III	15	9127	0.037	1.89	1.03–3.49
Reduced mouth opening	2	1283	NS	1.71	0.42–7.04
Reduced TMD	7	3137	0.025	2.63	1.19–5.86
Abnormal neck extension	2	1137	NS	1.94	0.47–7.98
Abnormal teeth	21	2911	< 0.001	12.55	7.18–21.95
Grade of intubation ≥3	9	948	< 0.001	7.25	3.40–15.45
MacGrath usage	10	2740	0.022	2.51	1.24–5.09
DLT usage	3	686	NS	2.65	0.82–8.58

*TMD*  thyromental distance

*P* < 0.05 = significant

Anaesthetic risk factors include McGrath MAC usage (OR 2.51) and a Cormack and Lehane grade of 3 or more (OR 7.25), both of which were statistically significant. However, whether the case was elective or emergency was not significant.

There was a moderate positive linear association between age and ASA status *R* = 0.431 (*p* = 0.002) and no significant positive linear association between age and poor dentition *R* = 0.187 (*p* = 0.202).

**Fig. 1:**
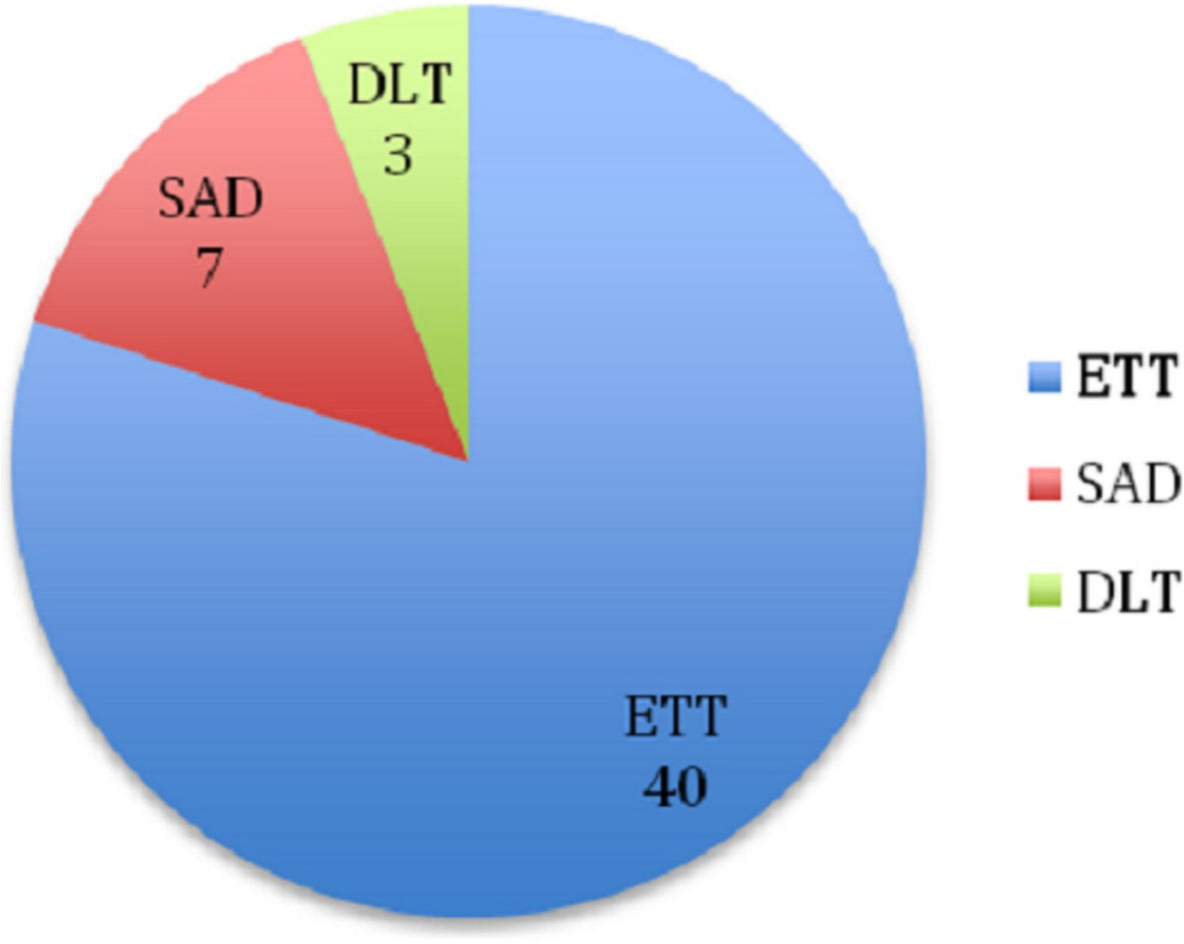
Distribution of airway devices used in patients with dental injury. Methods: This is a breakdown of the number of each type of airway device that was used in the patients who had sustained dental injury. Results: There were 40 ETTs, 7 SADs and 3 DLTs used. Figure definitions and descriptions: ETT = endotracheal tube, SAD = supraglottic aiwary device, DLT = double lumen tube

Figure 1 shows the distribution of types of airway devices used in the patients who suffered dental trauma. There were 7 cases of dental injury for patients who had SADs (0.029% of total patients who had SADs used). That contributed to 13.7% of the patients with dental trauma. This is in relation to a background rate of 43.2% SAD use in all cases done under general anaesthesia without dental trauma. Of the 7 cases with dental injury with use of SADs, 5 (71.4%) were had ProSeal LMA used, 1 (14.3%) had Supreme LMA used and 1 (14.3%) had I-gel use.

44 cases (86.3%) of dental injuries were discovered in the operating theatre (OT), followed by 4 cases (7.8%) in the post-anaesthetic care unit (PACU) and 1 (2%) in the ward. Of those with descriptive data, laryngoscopy was the most common cause of dental injury, occurring in 21 (41.2%) patients. This was followed by 4 (7.8%) cases during extubation, often accompanied by biting on the bite block during emergence, 3 (5.9%) cases during insertion of oral airway and 2 (3.9%) respectively during insertion of SAD and removal of SAD. 2 (3.9%) patients had their loose teeth removed by the anaesthetist prior to airway instrumentation. Other situations, with one case described each, were during mask ventilation without oral airway, during positioning, jaw thrust and by an otolaryngology surgeon intraoperatively. Unfortunately, location of dental injury was often poorly documented with only 23 of 51 patients documented. Upper right and left incisors were the most common locations of injury, with each having 7 patients. 3 cases were of the lower left incisor, and 1 each at the right upper canine, premolar, right lower incisor, canine, and left lower canine and premolar.

26 (51%) cases were not referred to dental services while 23 (45.1%) were referred. Of the cases referred to dental services, the majority, 13 (56.5%) did not have any immediate intervention and were given outpatient follow-up appointments. The commonest reason cited was pre-existing periodontal disease that rendered the dislodged teeth not implantable. 2 (8.7%) patients had dental extraction done and one patient had respectively, a chipped fragment and a crown cemented. Another patient had splinting done to a tooth that was laterally subluxed.

## Discussion

The rate of dental trauma associated with general anaesthesia in our institution is comparable to most centers. It is also not surprising that the most significant risk factor for dental injury is pre-existing dental abnormality, with a 12-fold increased risk. This emphasizes the need for appropriate risk counseling and adequate pre-operative dental management of diseased teeth.

Interestingly, if an anaesthetist anticipates difficulty with airway manipulation, it is 3 times more likely to have associated dental injury. This, coupled with other risk factors like reduced thyromental distance, and a Mallampati score more than 3 should increase the vigilance of the anaesthetist, in addition to planning for a difficult airway. This association is further illustrated by a 7-fold increased dental trauma risk associated with a Cormack and Lehane score ≥ 3. Age and ASA status correlated moderately which is not unexpected as co-morbidities are often associated with the aging population. However, our correlation studies did not show a significant positive correlation between age and poor dentition which is slightly unexpected and thus we may want to evaluate each individual’s dentition independent of their age groups when assessing dental risks.

The proportion of patients who had SADs used in the dental trauma population is significantly less than what would be expected given the widespread use of SADs in the general anaesthesia population. However, study has also shown that usage of a SAD does not obliterate dental trauma risk as a number of cases were still associated with SAD usage. Although insertion of a SAD often eliminates the risk associated with instrumenting the airway with a laryngoscope, the act of inserting a bulky device into the oral cavity still poses a risk especially if additional manipulation is required in a crowded airway. In addition, there may be involuntary biting on the hard stems of SADs during emergence that will not be preventable with careful insertion techniques that will result in dental injury in teeth at risk.

Videolaryngoscopy with McGrath MAC is associated with a two and a half times increased likelihood of dental injury, which was not a previously associated risk factor in older studies. Notably, the McGrath MAC was the videolaryngoscope most easily available in the institution at the time of review and the MAC was the only type of McGrath available. It is possible that this association is due to the increased prevalence of videolaryngoscopes used in daily practice for teaching purposes and increased availabilitiy. Also videolarygoscopes may have been used when increased risk of dental trauma was anticipated in order to mitigate the risk of dental injury during a potentially more hazardous laryngoscopy. However, the act of laryngoscopy may also be a factor in causing dental injury. 5.9% of our dental injury cases are associated with double lumen tube usage. Intuitively, insertion of the larger caliber double lumen tube may necessitate a larger mouth opening during laryngoscopy and that may increase the risk of dental trauma. Follow up studies may be needed to establish this risk factor.

Although data was incomplete, the location of most dental injuries was at the upper anterior region, which was consistent with other studies [2]. Surprisingly, half of the patients with dental injury did not have any dental referral or documentation of a referral. We can develop department guidelines on high dental risk management as well as a post injury protocol. This would ensure more consistent patient management, reducing incidence of perioperative dental injury, patient dissatisfaction at last minute cancellations and reducing medico-legal implications. Clear indications for urgent dental referral should be recommended, along with guidelines for proper handling of dislodged teeth. For example, patients with pre-existing periodontal disease with dental caries, or damaged deciduous dentition do not require urgent referral, as backed by our data suggesting no necessary immediate dental salvage procedures in most cases. Additionally, permanent tooth displaced from its socket can be stored in cool, fresh milk or normal saline until it can be splinted or fixed back in place.

This is a retrospective observational study only eliciting associations rather than causation. In addition, due to the retrospective nature of the study, some information were not well documented, for example, the experience of the person intrumenting the airway as well as exact data of the incidence of videolaryngoscopy in all cases. As such, we cannot establish possible risk factors such as inexperience and we had to use a surrogate of the number of MacGrath blades used as the incidence of videolaryngoscopy in the control group. Dental trauma data is only available via a self-reporting system and there may under-reporting. However, as dental injury reduction has been strongly promoted as a department clinical indicator, awareness of the problem amongst anaesthetists is high and that may increase reporting rates. Another limitation is that the total number of videolaryngoscope attempts could have been overestimated as blades could have been wasted. However, this will only result in a higher OR than obtained in our study as the incidence of dental injury with McGrath usage would be underestimated.

Future studies can be done on the background of this paper prospectively, to detect differences in incidences of dental injury using videolaryngoscopy, comparing at risk and normal risk patients. Videolaryngoscopy technique can also be reviewed to determine if it poses an increased risk of dental injury to patients. This is especially so as it has been shown that pressure exerted on a manikin with a videolaryngoscope (glidescope) was lower than that with a Macintosh blade and with lower pressure, it would be instuitive to conclude that risk of dental trauma would be lower [9]. With the advent of different videolaryngoscopes as well as different blades (hyperangulated and Macintosh-like), it would also be useful to compare the differences, if any it makes to potential dental trauma. Knowledge of the associated risk factors would promote awareness and lead to anaesthesia risk counseling as well as vigilance to avoid dental injury during airway manipulation.

## Conclusion

This study brings intio focus the importance of anticipating a traumatic airway manipulation for taking measures to alleviate the risk. Although usage of SADs have greatly reduced the need for intubation and thus reduced potential risks to dental injuries, it does not eliminate the risk completely and caution is still advised when inserting the deivces. Videolaryngoscopy should also be done with care and appropriate technique as it is often in at-risk patients that this device is utilised. It also highlights the importance of proper documentation, in terms of positioning of injury, circumstances of injury and further follow-up which has been often times ommitted. This is pertinent in developing preventive steps as well as for medicolegal purposes.

## Abbreviations

ASA: American Society of Anesthesiologists
DLT: Double lumen tube
LMA: Laryngeal Mask Airway
NUH: National University Hospital
OR: Odds Ratio
OT: Operating Theatre
PACU: Post Anaesthetic Care Unit
SAD: Supraglottic Airway Devices

## References

[1] Warner ME, Benenfeld SM, Warner MA, Schroeder DR, Maxson PM (1999). Perianesthetic dental injuries: frequency, outcomes, and risk factors. Anesthesiology.

[2] Newland MC, Ellis SJ, Peters KR, Simonson JA, Durham TM, Ullrich FA, Tinker JH (2007). Dental injury associated with anesthesia: a report of 161,687 anesthetics given over 14 years. J Clin Anesth.

[3] Lockhart PB, Feldbau EV, Gabel RA, Connolly SF, Silversin JB (1986). Dental complications during and after tracheal intubation. J Am Dent Assoc.

[4] Owen H, Waddell-Smith I (2000). Dental trauma associated with anaesthesia. Anaesth Intensive Care.

[5] Givol N, Gerhtansky Y, Halamish-Shani T, Taicher S, Perel A, Segal E (2001). Perianesthetic dental injuries: analysis of incident reports. J Clin Anesth.

[6] Chen JJ, Susetio L, Chao CC (1990). Oral complications associated with endotracheal general anesthesia. Anaesth Sinica.

[7] Mourão J, Neto J, Luís C, Moreno C, Barbosa J, Carvalho J, Tavares J (2013). Dental injury after conventional direct laryngoscopy: a prospective observational study. Anaesthesia.

[8] Milne A, Lockie J (2014). Dental damage in anaesthesia. Anaesth Intensive Care Med.

[9] Carassiti M, Zanzonico R, Cecchini S, Silvestri S, Cataldo R, Agrò FE (2012). Force and pressure distribution using Macintosh and GlideScope laryngoscopes in normal and difficult airways: a manikin study. Br J Anaesth.

